# Unusual perfusion patterns on perfusion-only SPECT/CT scans in COVID-19 patients

**DOI:** 10.1007/s12149-022-01761-5

**Published:** 2022-06-28

**Authors:** Bence Farkas, Zita Képes, Sándor Kristóf Barna, Viktória Szugyiczki, Magdolna Bakos, Attila Forgács, Ildikó Garai

**Affiliations:** 1 Scanomed Ltd. Nuclear Medicine Center Debrecen, Nagyerdei Boulevard Number 98, 4032 Debrecen, Hungary; 2 Department of Medical Imaging, Division of Nuclear Medicine and Translational Imaging, Faculty of Medicine, University of Debrecen, Nagyerdei Boulevard Number 98, 4032 Debrecen, Hungary; 3 Department of Nuclear Medicine, Békés County Pándy Kálmán Hospital, Semmelweis Street Number 1, 5700 Gyula, Hungary

**Keywords:** COVID-19, Perfusion-only SPECT/CT, Lung perfusion abnormalities

## Abstract

**Purpose:**

We aimed at examining both the incidence and extent of different lung perfusion abnormalities as well as the relationship between them on Tc-99m macroaggregated albumin (MAA) perfusion-only SPECT/CT scans in COVID-19 patients.

**Methods:**

Ninety-one patients (71.4 ± 13.9 years; range: 29–98 years, median age: 74 years; 45 female and 46 male) with confirmed SARS-CoV-2 virus infection were included in this retrospective study. After performing perfusion-only Tc-99m MAA SPECT/CT scans, visual, semi-quantitative assessment of the subsequent perfusion abnormalities was carried out: mismatch lesions (MM; activity defects on SPECT images identical to apparently healthy parenchyma on CT images), matched lesions (MA; activity defects with corresponding parenchymal lesions on CT scans), and reverse mismatch lesions (RM; parenchymal lesions with preserved or increased tracer uptake). Lesion-based and patient-based analysis were performed to evaluate the extent, severity, and incidence of each perfusion abnormality. Statistical tests were applied to investigate the association between the experienced perfusion impairments.

**Results:**

Moderately severe parenchymal lesions were detected in 87 (95.6%) patients. Although, 50 (54.95%) patients were depicted to have MM lesions, the whole patient cohort was mildly affected by this abnormality. MA lesions of average moderate severity were seen in most of the patients (89.01%). In 65 (71.43%) patients RM lesions were found with mild severity on average. Positive association was detected between total CT score and total RM score and between total CT score and total MA score. Significantly higher total CT scores were experienced in the subgroup, where RM lesions were present.

**Conclusions:**

Heterogeneous perfusion abnormalities were found in most of COVID-19 patients: parenchymal lesions with normal, decreased or increased perfusion and perfusion defects in healthy lung areas. These phenomena may be explained by the failure of the hypoxic pulmonary vasoconstriction mechanism and presence of pulmonary thrombosis and embolism.

## Introduction

Increasing attention is directed towards the pandemic coronavirus disease 2019 (COVID-19) caused by SARS-CoV-2 viral infection. Viral pneumonia, featured by hypoxia, is considered to be its most common manifestation [1]. Since assessment of early therapeutic response is mandatory to fight effectively against COVID-19-related hypoxia, there is an unmet need for the integration of sensitive non-invasive diagnostic methods into clinical routine with the aim of achieving better patient management.

Pulmonary embolism (PE) is one of the major complications of COVID-19. Based on recently conducted computed tomography (CT) studies, the incidence of COVID-19-associatied PE was detected to be between 22 and 30% [2, 3]. In addition, recent research suggests that besides alveolar damage other pathophysiological factors, such as microvascular pulmonary thrombosis might have a role in the development of hypoxia-induced COVID-19 pneumonia [4, 5].

CT pulmonary angiography (CTPA) has become the gold standard for the diagnosis of acute PE [6], but contraindications to intravenous contrast material (e.g., allergy to iodine-based contrast agents or severe renal failure) may preclude patients from undergoing the examination [7]. In this case, planar ventilation/perfusion (V/Q) scan, V/Q single photon emission computed tomography (SPECT) either combined with low-dose CT (ldCT), or Q-only SPECT/CT may be an alternative. However, due to the COVID-19 pandemic, safety concerns have risen about the routine V/Q protocols, as patients suspected of having PE has overlapping symptoms with individuals with COVID-19 infection, and during the ventilation part of the studies, aerosolized secretions are generated as a result of leakage, aerosolization, and coughing [8]. Besides the possible direct viral exposure of the personnel, these aerosolized secretions can contaminate the camera and imaging suite. Finally, performing ventilation scans in patients with poor clinical condition is challenging and the result is often unsatisfactory. These concerns have led to the recommendations to consider avoiding the ventilation scans during the evaluation of PE [8, 9]. Nevertheless, alternative methods have already been developed to replace the ventilation scans by the addition of low-dose CT to the perfusion study. According to the literature, these methods have similar or better diagnostic performance than the routine V/Q protocol [9, 10]. Thus, it is suggested to perform a Q-only SPECT combined with ldCT instead of the routine V/Q protocol in patients with suspicion of acute PE, if CTPA is contraindicated, as the ldCT component increases the diagnostic accuracy of the test as perfusion defects can be correlated with parenchymal abnormalities in the CT images [9]. To date, some articles have demonstrated that Tc-99m macroaggregated albumin (Tc-99m MAA) Q-SPECT/CT is a safe procedure without the increased risk of spreading contagious aerosols and enables to diagnose PE in patients with COVID-19, if contrast enhanced CT is contraindicated [11, 12]. In addition to identifying PE, Q-SPECT/CT examinations could detect other functional and morphological alterations in COVID-19 patients (e.g., infiltrates with increased perfusion), which may contribute to the better understanding of the pathomechanism of the disease [12].

Reviewing the literature, no research is available at the time of writing this manuscript which describes all abnormalities seen on Q-only SPECT/CT scans in a larger patient population suffering from active COVID-19. Few studies examined larger COVID-19 patient populations using lung perfusion scintigraphy but these articles were limited to evaluate only mismatch lesions caused by pulmonary embolism. The rest of the research on a similar topic is typically small-number case studies or methodological articles. The aim of the present study was to investigate the incidence of parenchymal lesions and different lung perfusion abnormalities and the relationship between them on Tc-99m MAA Q-SPECT/CT scans in 91 patients.

## Materials and methods

### Study participants

Q-SPECT/CT images of 91 participants (71.4 ± 13.9 years; range: 29–98 years, median age: 74 years) with positive diagnosis of SARS-CoV-2 virus infection and clinical symptoms suspicious of PE were evaluated in this single-center, retrospective study. The examinations were performed between 19/11/2020 and 07/01/2021. Forty-five (49.5%) of the patients studied were female and 46 (50.5%) were male. Reviewing the patient history, 6 (6.6%) of the selected individuals had a history of COPD and 3 (3.3%) of them had emphysema. Asthma has been reported in 11 (12.1%) of the included patients. Only 5 (5.5%) patients had a history of smoking. Due to the low number of cases in each group, subgroup analyzes were not performed. The indications of the examination included clinical deteriorations and symptoms characteristic for PE. Inclusion criteria were as follows: available Q-SPECT/CT images, confirmed diagnosis of COVID-19 maximum 2 weeks prior to the examination or clinically suspected COVID-19, which was laboratory confirmed by positive real-time polymerase chain reaction (RT-PCR) test within a week after the Q-SPECT/CT examination.

COVID vaccines were not yet available at the time of SPECT/CT studies; therefore, none of the patients was immunized. All procedures followed were in accordance with the ethical standards of the responsible university authority on human experimentation (DE RKEB/IKEB: 5916–2021).

### Acquisition protocol

Five minutes after the intravenous injection of 200 MBq (5.4 mCi) of Tc-99m MAA (Macro-Albumon, Medi-radiopharma Ltd., Érd Hungary) in supine position, acquisition started applying Anyscan SPECT/CT/PET (Mediso Ltd., Budapest, Hungary) system. Low-dose CT scans of the chest were recorded during free-breathing at 120 kV, 50–150 mAs without intravenous contrast administration, while SPECT image parameters of the same region were the following: 64 views, 30 s/step and 128 matrix size. Q-SPECT images were reconstructed utilizing ordered subset expectation maximization reconstruction, then fused with the corresponding CT image slices. The examinations were performed according to the protocol prepared by the infection control department of the hospital.

### Image interpretation and image processing

Selected Q-SPECT/CT scans were presented randomly and retrospectively reviewed by two nuclear medicine physicians with 6 and more than 20 years of experience. In case of discordance between readers, a simultaneous reading to reach consensus was achieved. Neither the clinical status of the patients nor the previous interpretation of the examinations was revealed to the evaluators. SPECT, CT and fused images were interpreted simultaneously using InterView Fusion software (version: 3.08.009.0000; Mediso Ltd., Budapest, Hungary).

The presence and the extent of the following CT morphological abnormalities, presumably caused by COVID-19, were evaluated: ground glass opacities (GGOs), consolidations, and mixed lesions. No distinction was made between the aforementioned lesions during the assessment.

**Fig. 1:**
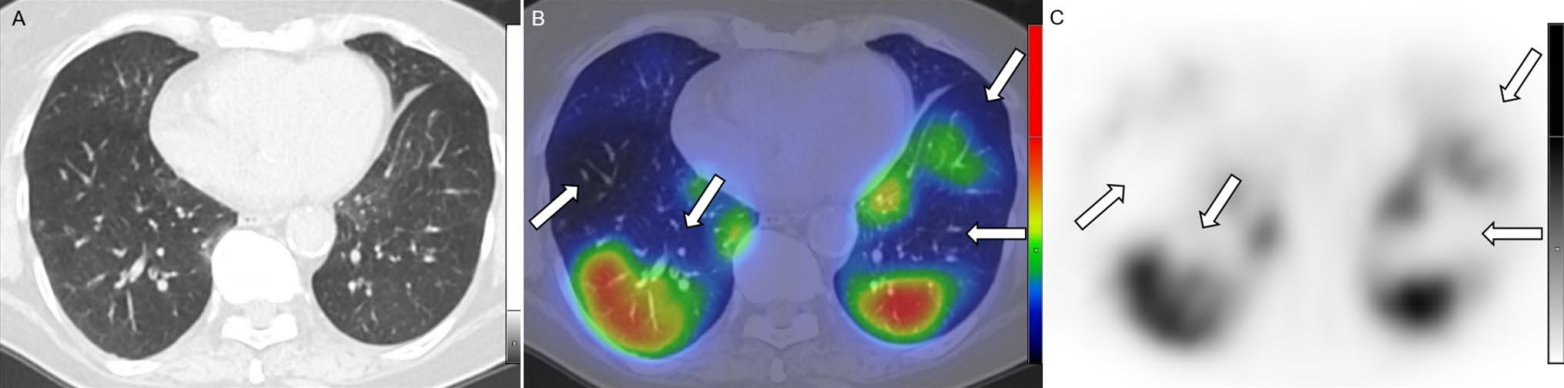
Axial low-dose CT (**a**), fused Q-SPECT/CT (**b**), and SPECT images (**c**) of a COVID-19 patient with dyspnea. A representative figure of bilateral segmental and subsegmental perfusion defects (demonstrated with white arrows) without any ventilation abnormalities. No pulmonary opacities could be depicted in this case (total CT score was 0). *CT* computed tomography, *Q-SPECT/CT* perfusion single-photon emission computed tomography/computed tomography, *COVID-19* coronavirus disease 2019

**Fig. 2:**
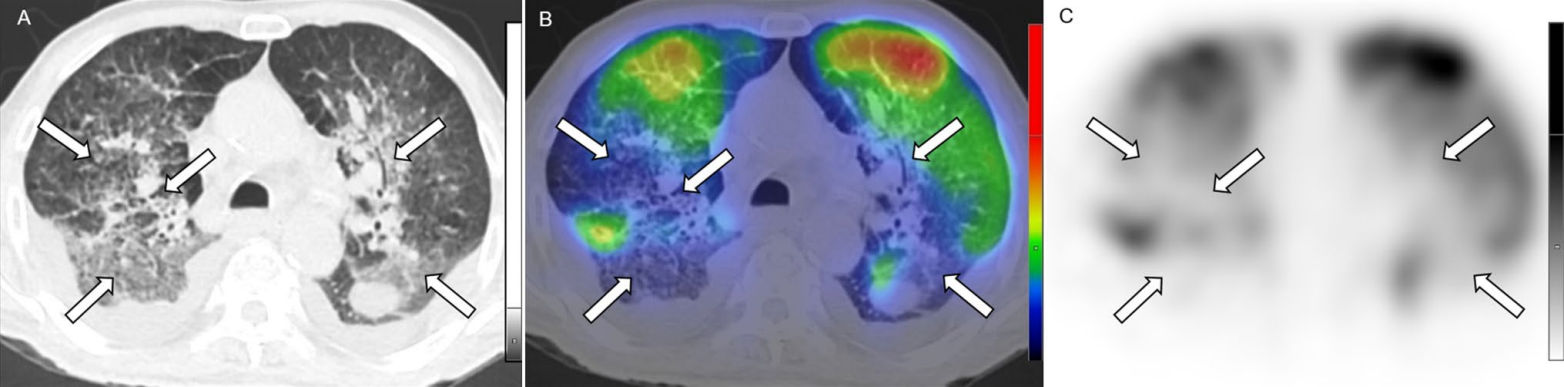
Axial low-dose CT (**a**), fused Q-SPECT/CT (**b**), and SPECT images of a COVID-19 patient. Extensive areas with decreased perfusion are detected in both lungs corresponding to ground-glass opacities and consolidations (as demonstrated *white arrows*). Bilateral, pleural effusion with no tracer accumulation could also be visualized in dorsal lung territories. Preserved pulmonary parenchyma shows normal tracer-distribution. *CT* computed tomography, *Q-SPECT/CT* perfusion single-photon emission computed tomography/computed tomography, *COVID-19* coronavirus disease 2019

**Fig. 3:**
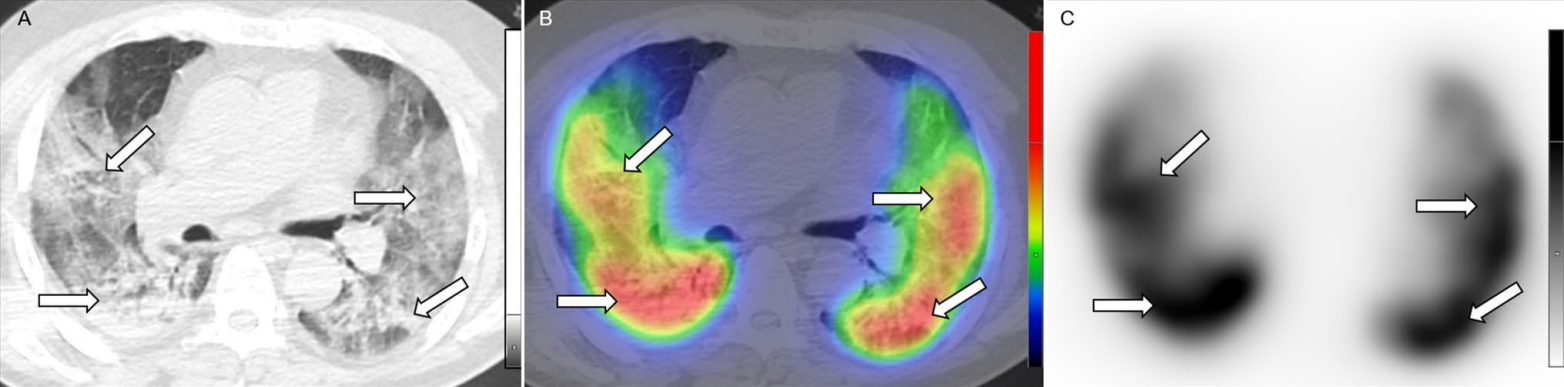
Axial low-dose CT (**a**), fused Q-SPECT/CT (**b**), and SPECT scans (**c**) of a COVID-19 patient. Fused SPECT/CT scan reveals remarkably extensive hyperperfused opacities in both lungs (shown with *white arrows*) compared to the relatively preserved lung areas. *CT* computed tomography, *Q-SPECT/CT* perfusion single-photon emission computed tomography/computed tomography, *COVID-19* coronavirus disease 2019

The presence and extent of the following perfusion abnormalities (denominated after the terms applied in V/Q scintigraphy) were evaluated on fused SPECT/CT scans:

1. Mismatch (MM) lesions: activity defects on SPECT images identical to apparently healthy pulmonary parenchyma on CT images (Fig. 1.).
2. Matched (MA) lesions: activity defects with corresponding parenchymal lesions on CT scans (Fig. 2**.**).
3. Reverse mismatch (RM) lesions: pulmonary parenchymal lesions with preserved or increased perfusion (Fig. 3.).

### Lesion-based analysis

\Visual, semi-quantitative assessment of the extent of the lesions was performed using a scoring system, based on a subtraction CT angiography study [13]. First, both lungs were divided into five lobes: right upper (RUL), middle (RML), lower lobe (RLL), and left upper (LUL), lower lobe (LLL).

With the aim of determining the extent of anatomic involvement (CT score) on low-dose CT scans, each lobe was scored from to 0–3 points as shown in Table 1. Then, individual lobar scores were generated to get the total lung CT severity score (total CT score), ranging from 0 to 15 points.

**Table 1:** Scoring of the severity of parenchymal involvement and different perfusion abnormalities in the lung lobes

Lobar CT score	
0	No parenchymal involvement in the lobe
1	≤ 30% parenchymal involvement in the lobe
2	31–60% parenchymal involvement in the lobe
3	> 60% parenchymal involvement in the lobe
Lobar mismatch (MM), matched (MA), or reverse mismatch (RM) score	
0	The particular phenomenon is not detected in the lobe
1	The particular phenomenon affects ≤ 50% of the lobe
2	The particular phenomenon affects > 50% of the lobe

To determine the extent of anatomic involvement (lobar CT score) 0–3 points were given for each lobe. In the case of perfusion abnormalities, seen in Q-SPECT/CT images, each phenomenon (lobar MM score, lobar MA score, and lobar RM score) was given 0–2 points per lobe, separately

In case of perfusion abnormalities, seen on Q-SPECT/CT images (MM, MA, and RM lesions), each phenomenon was given 0–2 points per lobe, separately. The extent of each particular lesion in each lobe was graded as shown in Table 1. The total MM, total MA or total RM score is the sum of each lobar MM, MA or RM scores, respectively, ranging from 0 to 10 points separately.

### Patent-based analysis

The incidence of each lesion (CT parenchymal abnormality, MM lesions, MA lesions, and RM lesions) in the overall patient population was assessed separately. Lesions were considered to be presented if the respective total score of the particular lesion was 1 or higher in a given patient.

To stratify the total parenchymal involvement, patients were divided into three subgroups based on total CT scores (ranging from 0 to 15). Parenchymal involvement was either considered mild, moderate or severe, if the total CT scores were between 1 and 4; 5 and 9; or  ≥ 10, respectively.

Based on each perfusion abnormality seen on fused Q-SPECT/CT scans, subjects were further categorized into three–three severity groups. Using the total MM, total MA, and total RM scores (each ranging from 0 to 10), the severity of the particular perfusion abnormality was either considered mild, moderate or severe if the respective total scores were between 1 and 3; 4 and 6; or > 7, respectively.

### Statistical analysis

The statistical analysis was performed with IBM SPSS Statistics 26 software package (SPSS Inc.). Nonparametric Spearman’s rank-order correlation was calculated to measure strength and direction of association between ordinal variables (CT, MM, MA, RM). Chi-Square test was used to determine the co-occurrence of RM and MM lesions in the population. Nonparametric Mann–Whitney *U* test was performed to measure the difference in CT score in case the examined population was divided into subgroups based on the presence or lack of MM or RM lesions. The same statistical test was performed to examine the difference between total MM score and total MA score depending on the presence of RM deviation.

## Results

### Lesion-based analysis

The average and median total CT severity scores (out of the maximum of 15 points) of the examined patients were 6.91 and 7, respectively, which means that overall, moderately severe parenchymal lesions could be depicted in the study population. On the ldCT scans, the most severe parenchymal involvement was detected in the lower lobes, while the RUL, LUL, and RML were less affected (demonstrated in Table 2.).

**Table 2:** Average lobar CT, lobar mismatch (MM), lobar matched (MA), and lobar reverse mismatch (RM) severity scores in the study population

Lobe	Lobar CT score^a^	Lobar MM score^a^	Lobar MA score^a^	Lobar RM score^a^
Right upper lobe	1.33	0.51	0.85	0.42
Right middle lobe	0.99	0.33	0.53	0.18
Right lower lobe	1.77	0.25	0.95	0.75
Left upper lobe	1.27	0.32	0.78	0.48
Left lower lobe	1.55	0.26	0.85	0.58

^a^Lobar CT scores range between 0 and 3 points, the lobar MM, MA, and RM scores range between 0 and 2 points

The average and median total MM scores (out of a maximum of 10 points) were only 1.67 and 1, respectively, meaning that these lesions mildly affected the entire study population. If the subpopulation having the lesion is examined separately, the average and median total MM scores are 2.94 and 3, respectively, which indicates mild mean severity even in the affected subpopulation. The RUL was most commonly (*n* = 36, 39.56%), and the RLL was the least frequently (*n* = 17, 18.68%) affected by MM lesions. However, it is worth highlighting that in case of 27 patients (29.67%) at least one MM lesion affecting more than 50% of the lobe was detected. In the presence of MM lesions multiple lobe involvement could be assessed.

The average and median total MA scores (out of the maximum of 10 points) were 3.95 and 4, respectively, meaning that the mean severity of these lesions was moderate in the entire study population. Similar values were observed if we examined only the affected subpopulation (average total MA score: 4.43; median total MA score: 4). We found that the RLL showed the highest average MA score, and the RML had the lowest average MA score, similarly to CT severity scores. MA lesions affecting more than 50% of a lobe were present in 29 patients (31.87%) and in 61 lobes.

The average and median total RM scores (out of the maximum of 10 points) were 2.41 and 1, respectively, meaning mild severity in the studied population. The mean severity remained mild in the subpopulation affected by RM lesions (average total RM score: 2.94; median total RM score: 2.5). RM lesions were most commonly detected in the RLL (in 48 cases). The RLL showed the highest, while the RML had the lowest average RM score. RM lesions affecting more than 50% of a lobe were present in 23 patients (25.27%) and in 47 lobes. In a few cases, these lesions had markedly higher uptake compared to healthy lung areas.

We found significant positive association between total CT score and total RM score (Spearman’s rho: 0.626,* p* < *0.001*) and between total CT score and total MA score (Spearman’s rho: 0.728,* p* < *0.001*). Total MM score showed no correlation with either total CT score, total MA score, or total RM score. No significant correlation was found between total MA score and total RM score (Spearman’s rho: 0.096,* p* = *0.363*)*.* No association was found either between the co-occurrence of RM lesions and MM lesions (*p* = *0.687*)*.* Utilizing Mann–Whitney *U* probe, total CT scores were detected significantly higher in the subgroup, where RM lesions were present, although no significant difference was observed between the two groups regarding total MA and total MM scores. When patients were classified based on the presence of MM lesions, the total CT, RM, and MA scores did not differ significantly between the two groups.

### Patent-based analysis

Lung involvement was presented in the form of pure GGOs, consolidations, and mixed lesions with GGO or consolidation predominant pattern on the ldCT scans. Any of the former parenchymal lesions was detected (total CT score ≥ 1) in 87 out of the 91 patients (95.6%). Mild parenchymal involvement (total CT score: 1–4 points) was observed in 24 (26.37%) patients, moderate (total CT score: 5–9 points) in 37 (40.66%) and severe (total CT score: 10–15 points) in 26 (28.51%) involved subjects. Further analysis of the different morphological patterns and their associations with clinical features was beyond the scope of the present study.

Perfusion defects without obvious parenchymal lesions (MM lesions) were detected in 50 out of 91 patients (54.95%). However, it should be noted, that the occurrence of this abnormality could not be evaluated in case of 3 patients, as no healthy parenchyma could be differentiated in the lungs on the ldCT images due to extensive diffuse GGOs and consolidations. In contrast, on another 3 (3.3%) Q-SPECT/CT scans, perfusion defects were detected without parenchymal involvement (the total CT score was 0) neither in the region of the perfusion defects nor in other areas of the lungs (Fig. 1.). In case of the detailed abnormality, mild involvement (total MM score: 1–3 points) was observed in the majority of the enrolled patients (*n* = 34; 37.36%), while moderate involvement (total MM score: 4–6 points) was found in 14 (15.38%), and severe involvement (total MM score: ≥ 7 points) was detected in only 2 (2.2%) patients.

Perfusion defects with corresponding CT morphological abnormality (MA lesions) were seen in 81 out of the 91 cases (89.01%). Regarding MA lesions, the majority of the subjects could be characterized by either moderate (total MA score: 4–6 points; n = 32; 35.16%) or mild (total MA score: 1–3 points; *n* = 30; 32.97%) involvement, 19 (20.88%) patients showed severe exposure (total MA score ≥ 7 points).

A third type of perfusion abnormality, appearing in 65 out of 91 patients (71.43%) was also observed, with preserved or increased tracer accumulation in the injured lung parenchyma (RM lesions). This alteration most often had mild effects (total RM score: 1–3 points; *n* = 40; 43.96%); however, moderate involvement (total RM score: 4–6 points) was observed in 16 cases (17.58%), and severe (total RM score: ≥ 7 points) in 9 cases (9.89%).

## Discussion

This study investigated the incidence of different lung perfusion abnormalities and the relationship between them on Tc-99m MAA Q-SPECT/CT scans in 91 SARS-CoV-2 infected patients with clinical suspicion of PE and contraindication to CTPA.

In the present study, areas of decreased activity in intact lung areas were detected in 54.95% of the patients and no connection was found between MM lesions and parenchymal involvement. It is important to note, that in COVID-19 pneumonia, pulmonary thrombosis can also cause perfusion defects in addition to PE, so the presence of MM lesions does not necessarily mean the diagnosis of PE [14]. In a dual-energy CT study, Grillet et al*.* [15] observed no significant difference in the incidence of parenchymal ischemia between patients with and without pulmonary artery thrombosis, indicating pulmonary vessel microthrombosis associated with COVID-19. This theory is supported by autopsy findings, as obstruction of pulmonary arteries by thrombotic material was present at both the macroscopic and microscopic level. As mainly arteries smaller than 1 mm in diameter were occluded, the authors considered that thrombosis was the more likely cause than embolism [16]. Nonetheless, increased incidence of PE was also reported in a significant proportion of COVID-19 patients [2, 3, 17], which can be a consequence of traditional risk factors in critically ill patients (e.g., immobilization, use of vasoactive drugs) and extremely activated clotting system [4, 14]. Summarizing the literature data, pulmonary microthrombi may primarily cause more distal and smaller perfusion defects, while hypoperfused areas caused by PE may be more central and larger [15, 16]. The distinction between pulmonary microthrombi and PE also have clinical relevance, because they require a different treatment strategy [14]. However, their separation on Q-only SPECT/CT images is beyond the scope of the present study.

In our examination, opacities with preserved or increased perfusion were frequently observed in the patients (71.43%) and the positive correlation between the total CT score and total RM score also assumes retained perfusion in some of the parenchymal lesions. The presence of normal or high pulmonary blood flow in non-aerated lung areas is probably caused by the relative failure of the hypoxic pulmonary vasoconstriction mechanism in COVID-19 pneumonia [13, 18]. Mathematical modeling has also confirmed that the amount of pulmonary shunt observed in patients with early stage severe COVID-19 is not plausible without hyperperfusion of the injured lung [19].

Pulmonary infiltrates with decreased blood flow were the most common perfusion abnormality, seen in 89.01% of the cases. The positive correlation found between total MA score and total CT score indicates that the number of hypoperfused lesions increased when more severe parenchymal involvement was detected.

**Fig. 4:**
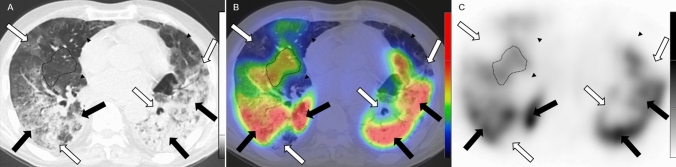
Axial low-dose CT (**a**), fused Q-SPECT/CT (**b**), and SPECT images of a COVID-19 patient. GGOs of variable size are represented in all pulmonary lobes, while extensive consolidations are present in the posterior areas on low-dose CT scans. A wide-variety of perfusion abnormalities could be depicted on fused Q-SPECT/CT scans: GGOs, consolidations with either decreased (*white arrows*) or increased (*black arrows*) perfusion, and subpleural perfusion defects in normally aerated lung parenchyma (*arrowheads*). Uninvolved lung territory with intact perfusion was marked with a dotted line. *CT* computed tomography, *Q-SPECT/CT* perfusion single-photon emission computed tomography/computed tomography, *COVID-19* coronavirus disease 2019, *GGO* ground-glass opacity

It should be mentioned, that in advanced cases, the perfusion of parenchymal abnormalities was very heterogeneous (Fig. 4.), which is substantiated by the fact that no association was found between total MA and RM score in the present study. Similarly to our findings, dual-energy CT and subtraction CT angiography studies also described marked heterogeneity in pulmonary perfusion in COVID-19 patients: maintained or increased perfusion in opacities and perfusion defects or hypoperfusion in normally ventilated lung areas were detected [13, 15, 20].

Regarding lobar involvement, the highest CT severity scores were observed in the lower lobes and the same lobes showed the highest MA and RM scores. These findings also suggest heterogeneous perfusion of the parenchymal lesions. In contrast, MM lesions mostly affected the right upper, middle, and the left upper lobes. These observation is consistent with literature data, as patients with pulmonary infiltrates show stronger perfusion in the non-affected lung areas, thus clots should be expected in vessels of non-affected pulmonary segments [9].

## Limitations

This study has limitations. First, it was a retrospective, monocentric study, which could lead to selection bias. This bias was limited by including consecutive patients. Second, as is known, the spatial resolution of SPECT is worse than CT, thus it is difficult to judge whether tracer accumulation is inside or right adjacent to smaller parenchymal lesions. Third, at the time of writing this study, no clinical information other than Q-SPECT/CT data sets and the results of PCR tests were available, which did not allow to assess the clinical relevance of the found abnormalities. For this purpose, further studies are required.

Finally, at the time of writing the manuscript, no study was available in which all abnormalities, observed on Q-only SPECT/CT scans in COVID patients, were quantified. Therefore, the scoring system used in our study is based on an article in which lung parenchyma and perfusion anomalies in COVID patients were examined by subtraction CT angiography [13]]. In this paper, parenchymal involvement seen on CT scans was scored between 0 and 3 points, while 0–2 points were used to describe the extent of hypoperfusion seen in intact lung areas. We assume that the same scoring method could have been used for SPECT lesions as used to assess parenchymal involvement seen on CT scans. However, the resolution of the two modalities is not the same, and incidental co-registration problems could make the evaluation of perfusion abnormalities more difficult. Thus, we believe that a different scoring system is appropriate, in which the individual severity groups can be more clearly distinguished from each other. We believe that the use of different scoring systems does not significantly affect the conclusions that can be drawn from our observations.

## Conclusions

In our Q-only SPECT/CT study heterogeneous perfusion abnormalities were found in most of the COVID-19 patients: parenchymal lesions were present with normal, decreased or increased perfusion and perfusion defects were frequently observed in healthy lung areas. These observed phenomena may be explained by the failure of the hypoxic pulmonary vasoconstriction mechanism and the presence of pulmonary thrombosis and embolism. Because our results are consistent with previous dual-energy CT and subtraction CT angiography studies, we hypothesize that Q-only SPECT/CT may be a useful tool in the evaluation of COVID-19 patients in whom CT contrast agent is contraindicated.
